# Adjunctive Cilostazol Therapy for Sinus Node Dysfunction After Heart Transplantation

**DOI:** 10.1016/j.jaccas.2026.109122

**Published:** 2026-07-02

**Authors:** Kento Tamano, Sakae Takenaka, Toshiyuki Nagai, Toshihisa Anzai

**Affiliations:** Department of Cardiovascular Medicine, Faculty of Medicine and Graduate School of Medicine, Hokkaido University, Sapporo, Japan

**Keywords:** atrioventricular junctional rhythm, cilostazol, heart transplantation, sinus node dysfunction

## Abstract

**Background:**

Adjunctive therapy with cilostazol, a phosphodiesterase-3 inhibitor, for sinus node dysfunction (SND) after heart transplantation (HTx) remains unestablished.

**Case Summary:**

We report the case of a 33-year-old woman who developed an atrioventricular junctional rhythm after HTx. Her SND had persisted for more than 3 weeks post-HTx despite treatment with positive chronotropic agents, including isoproterenol and tulobuterol. After the initiation of off-label cilostazol, her heart rate gradually increased. She was discharged without requiring permanent pacemaker implantation.

**Discussion:**

This is the first reported case describing the addition of cilostazol to a nonselective β-agonist and a β_2_-adrenergic agonist in a patient with atrioventricular junctional rhythm after HTx. In our case, the addition of cilostazol appeared to improve right ventricular function and cardiac index by increasing the heart rate without adverse events.

**Take-Home Message:**

Cilostazol may represent an adjunctive option for managing severe SND in heart transplant recipients.

## Visual Summary

**Figure:**
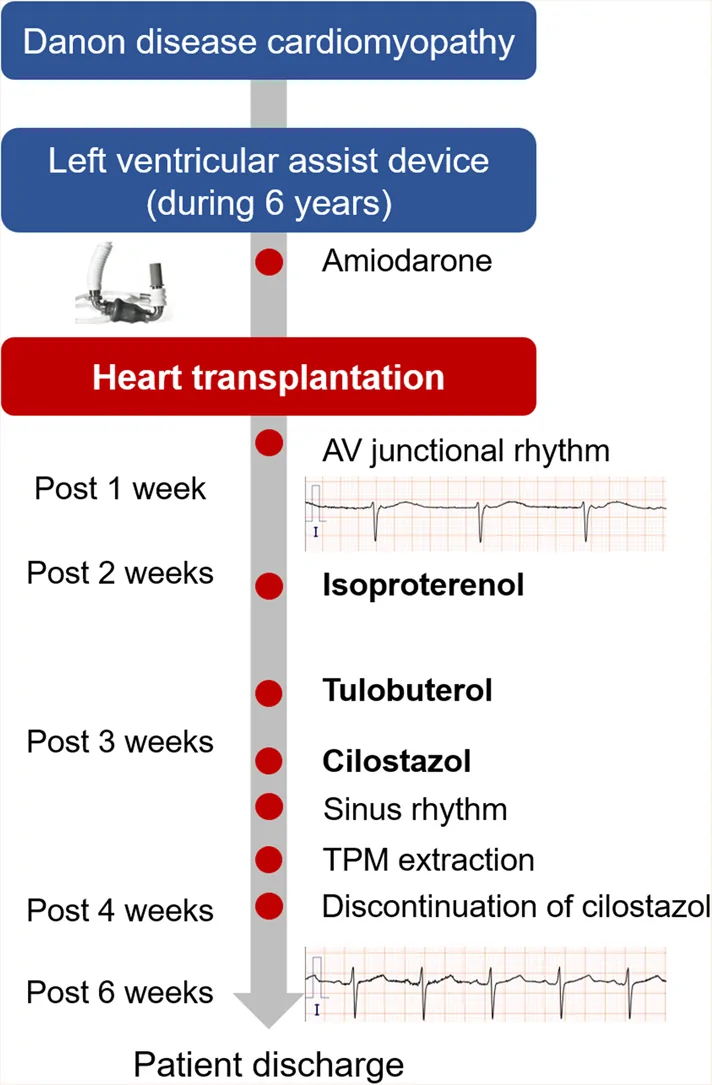

## History of Presentation

A 33-year-old woman with Danon disease–related cardiomyopathy presented with worsening heart failure and underwent implantation of a left ventricular assist device (LVAD). Amiodarone administration was continued during LVAD support because of ventricular tachycardia. She underwent heart transplantation (HTx) using a modified bicaval technique after 6 years of LVAD support. Atrioventricular (AV) pacing was performed through epicardial leads, and a heart rate (HR) of 80 to 100 beats/min was maintained in the early post-HTx period. Immunosuppressive therapy with a standard regimen of tacrolimus, mycophenolate mofetil, and prednisolone was initiated. The donor was a woman in her 40s with no history of cardiac disease. The HR of the donor had a normal sinus rhythm of approximately 70 beats/min before harvest. The total ischemic time was 245 minutes.

**Take-Home Message**

• The addition of cilostazol to both a nonselective β-agonist and a β_2_-adrenergic agonist may represent a therapeutic option for heart transplant recipients with severe sinus node dysfunction and could be considered before permanent pacemaker implantation.

## Past Medical History

The patient had received a diagnosis of Danon disease on the basis of clinical symptoms and the results of a lysosome-associated membrane protein 2 gene mutation. Despite guideline-directed medical therapy and cardiac resynchronization therapy defibrillator, her heart failure worsened, and she underwent LVAD implantation as a bridge to transplantation. Four years after LVAD implantation, she developed severe aortic insufficiency, which required surgical aortic valve closure.

## Differential Diagnosis

Initial consideration was sinus node dysfunction (SND), which is a common complication in the early postoperative period after HTx, with a reported prevalence of up to 50%.^1^^,^^2^ However, it was necessary to exclude post-transplant complications, such as acute rejection and coronary artery disease.

## Investigations

**Figure 1:**
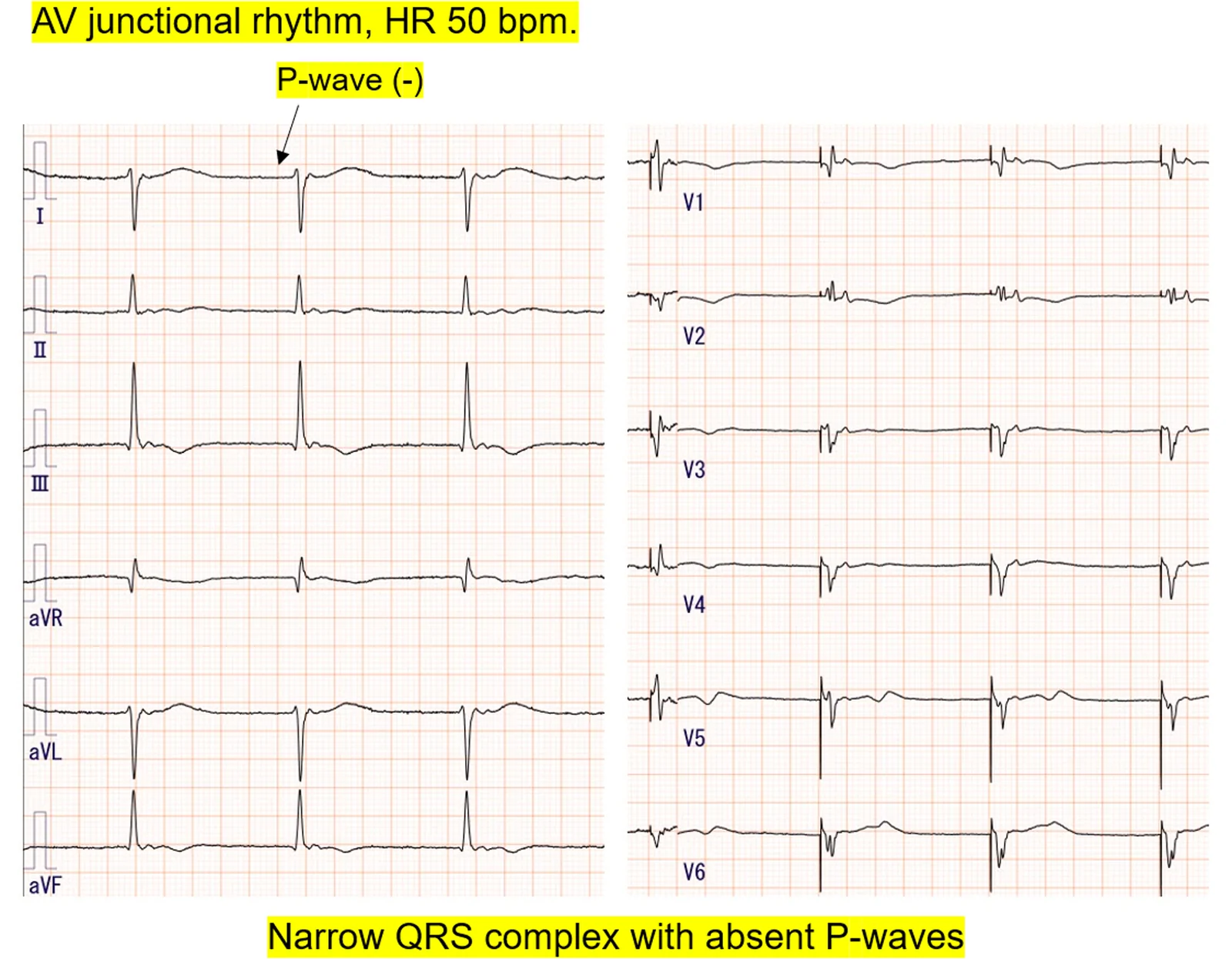
Electrocardiography Before the Initiation of Cilostazol Electrocardiography reveals a heart rate of 50 beats/min, with an AV junctional rhythm. AV = atrioventricular; HR = heart rate.

One week post-HTx, echocardiography showed a normal left ventricular ejection fraction of 65%, and her hemodynamic parameters on right heart catheterization were stable with AV pacing (Table 1). Endomyocardial biopsy revealed no evidence of rejection. However, electrocardiography (ECG) showed that the HR remained at approximately 50 beats/min, with an AV junctional rhythm when the pacing HR was lowered (Figure 1). She experienced shortness of breath and high levels of N-terminal pro–brain natriuretic peptide and total bilirubin (Table 2). The levels of amiodarone and desethylamiodarone remained high (amiodarone 615 ng/mL; desethylamiodarone 764 ng/mL) despite the discontinuation of amiodarone post-HTx.

**Table 1:** Hemodynamic Parameters Post-HTx

	Pre-Cilostazol Administration			Post-Cilostazol Administration	
	Post 1 wk	Post 2 wk	Post 3 wk	Post 4 wk	Post 6 wk
HR (beats/min)	80	80	50	90	85
Rhythm	Pacing	Pacing	AV junctional	Sinus	Sinus
Systolic arterial pressure (mm Hg)	101	107	105	120	110
Diastolic arterial pressure (mm Hg)	61	62	55	64	60
Mean arterial pressure (mm Hg)	74	69	71	82	78
RAP (mm Hg)	5	13	15	12	6
PAWP (mm Hg)	8	15	16	16	10
Systolic PAP (mm Hg)	27	42	38	37	27
Diastolic PAP (mm Hg)	10	15	14	14	12
Mean PAP (mm Hg)	16	25	21	23	19
PVR (Wood units)	2.1	2.0	1.5	1.5	2.2
RVSWI (mm Hg/L/m^2^)	4.5	6.5	3.3	5.3	5.6
PAPi	3.4	2.1	1.6	1.9	2.5
RAP/PAWP ratio	0.63	0.87	0.94	0.75	0.60
CO (L/min)	3.8	5.0	3.4	4.8	4.0
CI (L/min/m^2^)	2.4	3.2	2.2	3.2	2.7
SvO_2_ (%)	69	59	54	67	66

AV = atrioventricular; CI = cardiac index; CO = cardiac output; HR = heart rate; HTx = heart transplantation; PAP = pulmonary artery pressure; PAPi = pulmonary artery pulsatility index; PAWP = pulmonary artery wedge pressure; PVR = pulmonary vascular resistance; RAP = right atrial pressure; RVSWI = right ventricular stroke work index; SvO_2_ = mixed venous saturation.

**Table 2:** Laboratory Findings

	Pre-Cilostazol Administration			Post-Cilostazol Administration		
	Post 1 wk	Post 2 wk	Post 3 wk	Post 4 wk	Post 5 wk	Post 6 wk (at discharge)
WBC count (1,000/μL)	6.0	5.3	6.1	2.9	3.1	3.8
Hemoglobin (g/dL)	11.0	7.7	8.0	8.3	9.5	10.7
Total protein (g/dL)	5.6	5.9	6.0	6.1	6.4	6.5
Albumin (g/dL)	3.0	3.1	3.3	3.4	3.7	3.7
Total bilirubin (mg/dL)	3.9	4.0	3.1	4.1	3.0	1.8
AST (IU/L)	17	49	48	41	28	30
ALT (IU/L)	19	108	133	87	53	47
BUN (mg/dL)	23	270	13	11	14	14
Serum creatinine (mg/dL)	0.6	0.7	0.7	0.8	0.9	0.8
Serum sodium (mEq/L)	135	132	136	136	141	140
CRP (mg/dL)	0.56	0.60	0.40	0.05	0.04	0.48
NT-proBNP (pg/mL)	1,459	2,877	2,437	2,808	1,796	873
Tacrolimus trough levels (ng/mL)	4.3	7.3	7.9	12.9	12.6	10.2
Amiodarone; desethylamiodarone (ng/mL)		615; 764		538; 705	571; 681	433; 544

ALT = alanine aminotransferase; AST = aspartate aminotransferase; BUN = blood urea nitrogen; CRP = C-reactive protein; NT-proBNP = N-terminal pro–brain natriuretic peptide; WBC = white blood cell.

## Management

**Figure 2:**
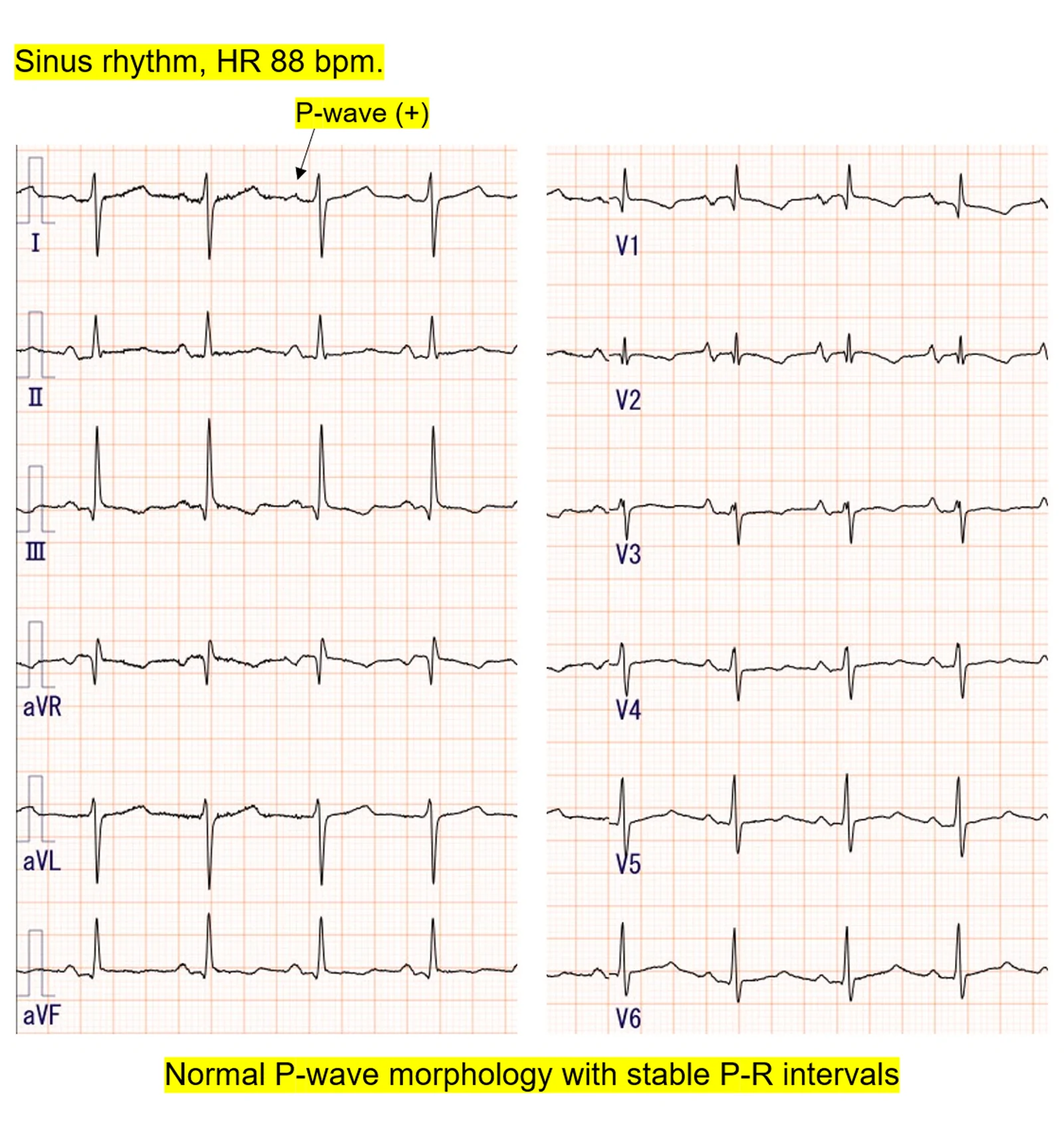
Electrocardiography After the Initiation of Cilostazol Electrocardiography reveals a heart rate of 88 beats/min, with sinus rhythm. HR = heart rate.

Two weeks post-HTx, the SND did not improve because of multiple factors, including pretransplant amiodarone administration. Therefore, we administered pharmacological chronotropic agents while awaiting recovery of normal sinus node function. Because she tended to experience strong palpitations when using intravenous drugs, oral administration of isoproterenol 45 mg/d, a nonselective β-agonist, was initiated first on postoperative day (POD) 16. AV junctional rhythm persisted even after initiating isoproterenol, and we added tulobuterol tape 2 mg/d, a β_2_-adrenergic agonist on POD 20. The ECG monitor repeatedly displayed sinus bradycardia and AV junctional rhythm, preventing the extraction of the epicardial leads. Three weeks post-HTx, right heart catheterization revealed elevated intracardiac pressure, low cardiac index, impaired right ventricular (RV) function, and decreased mixed venous saturation (Table 1). The left ventricular ejection fraction did not change, and endomyocardial biopsy showed no evidence of rejection. Coronary angiography revealed no significant arterial lesions. We considered permanent pacemaker (PPM) implantation; however, the recipient was already colonized with pathogens such as *Staphylococcus aureus* and viridans streptococci and was at high risk for device-related infection in her immunosuppressed state. Given these findings, on POD 23, we added the off-label use of cilostazol 200 mg/d, a phosphodiesterase-3 inhibitor, to maintain HR after obtaining appropriate informed consent. Although amiodarone and desethylamiodarone levels remained high (amiodarone 538 ng/mL; desethylamiodarone 705 ng/mL) (Table 2), the HR gradually increased to approximately 90 beats/min (Figure 2), and her hemodynamic status stabilized after the initiation of cilostazol (Table 1).

## Outcome and Follow-Up

**Figure 3:**
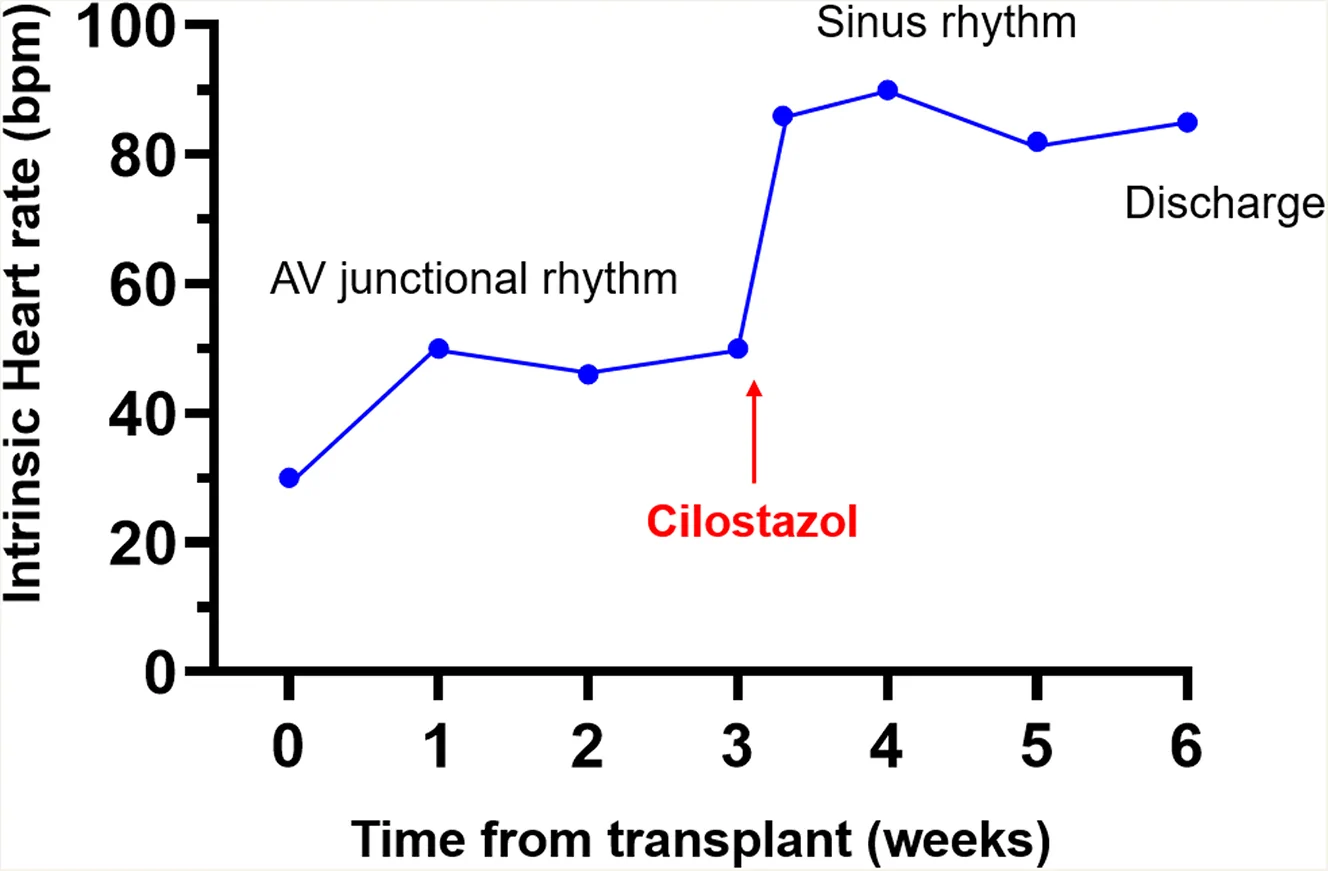
Changes in Intrinsic Heart Rate AV = atrioventricular.

Given that cilostazol carries potential risks, such as proarrhythmic effects, increased bleeding, and drug-drug interactions with immunosuppressive therapy, the patient underwent continuous ECG monitoring with close attention, and laboratory parameters, including hemoglobin and tacrolimus trough levels, were evaluated daily. After the initiation of cilostazol, premature atrial contractions were observed; however, no ventricular arrhythmias occurred. Although anemia was observed before cilostazol initiation, active bleeding was ruled out, and the anemia gradually resolved after linezolid, which was strongly suspected as the cause, was discontinued. Moreover, cilostazol did not significantly affect the concentration levels or doses of immunosuppressants. After confirming that the patient's HR remained stable, we tapered cilostazol to 100 mg/d on POD 27 and discontinued it on POD 28. After discontinuation, the HR remained stable at 80 to 90 beats/min with sinus rhythm, and laboratory data, including N-terminal pro–brain natriuretic peptide, total bilirubin, and amiodarone levels, also improved (Table 2). Therefore, the patient was discharged without the need for PPM implantation (Figure 3). Isoproterenol and tulobuterol were continued in the outpatient setting.

## Discussion

We present a rare case of AV junctional rhythm after HTx that was successfully treated with isoproterenol, tulobuterol, and cilostazol. To our knowledge, this is the first report demonstrating that the addition of cilostazol to both a nonselective β-agonist and a β_2_-adrenergic agonist was effective and safe in a heart transplant recipient with severe SND.

HTx is an established treatment for patients with advanced heart failure that is refractory to medical therapy and cardiac resynchronization therapy. Although long-term survival after HTx has continued to improve, the overall clinical benefit remains limited by postoperative complications. SND is very common in the early postoperative period after HTx and is believed to be multifactorial in origin, including surgical trauma, cardiac denervation, and ischemia-reperfusion injury.^3^ The biatrial anastomosis technique carries a higher risk of sinus node injury because of the proximity of right atrial suture lines, whereas the bicaval technique avoids this risk by anastomosing the left atrium with separate bicaval anastomoses rather than both atria. Previous reports have shown higher rates of SND and PPM implantation with the biatrial technique compared with the bicaval technique, leading to a shift in surgical practice.^4^ In addition, because amiodarone has a very long half-life, pretransplant amiodarone use is a well-established risk factor for sinus bradycardia. Recipients who receive amiodarone before HTx are at an increased risk of PPM implantation after HTx.^5^ Given the prolonged waiting time for HTx, the impact of long-term amiodarone administration on post-HTx outcomes can no longer be overlooked.

In the early postoperative period, cardiac allografts typically exhibit restrictive hemodynamic status, and a higher HR is required to maintain adequate cardiac output. AV pacing after HTx is usually achieved using epicardial leads to support HR. The 2023 International Society for Heart and Lung Transplantation guidelines recommend positive pharmacological chronotropic agents, including nonselective β-agonists (isoproterenol) or β_2_-adrenergic agonists (theophylline), to maintain HR while awaiting recovery of normal sinus node function,^6^ and PPM implantation is indicated if symptomatic bradycardia persists beyond 3 weeks after HTx.^7^^,^^8^ However, PPM implantation is associated with several disadvantages, including procedural complications, prolonged hospital stay, and increased costs. In particular, heart transplant recipients receiving immunosuppressive therapy are at an increased risk of infection. Moreover, the need for permanent pacing often resolves within months of HTx in many patients.^9^ Therefore, optimizing and combining pharmacological chronotropic agents as safely as possible during the early post-HTx period is crucial.

In our case, although several factors, including spontaneous recovery of sinus node function, washout of amiodarone, the cumulative effect of isoproterenol and tulobuterol, and postoperative recovery, were related to improvement in HR, the addition of cilostazol may have contributed to improving RV function and the cardiac index by increasing HR without adverse effects. Unlike other agents, cilostazol is a phosphodiesterase-3–inhibiting drug and increases the HR through the up-regulation of cyclic adenosine monophosphate in the myocardium and sinus node.^10^ This is particularly relevant in the early post-HTx period when increasing HR may facilitate unloading of a struggling RV. Given the potential for adverse events such as proarrhythmic effects and bleeding, cilostazol must be carefully adjusted while closely monitoring the HR.

**Visual Summary:**
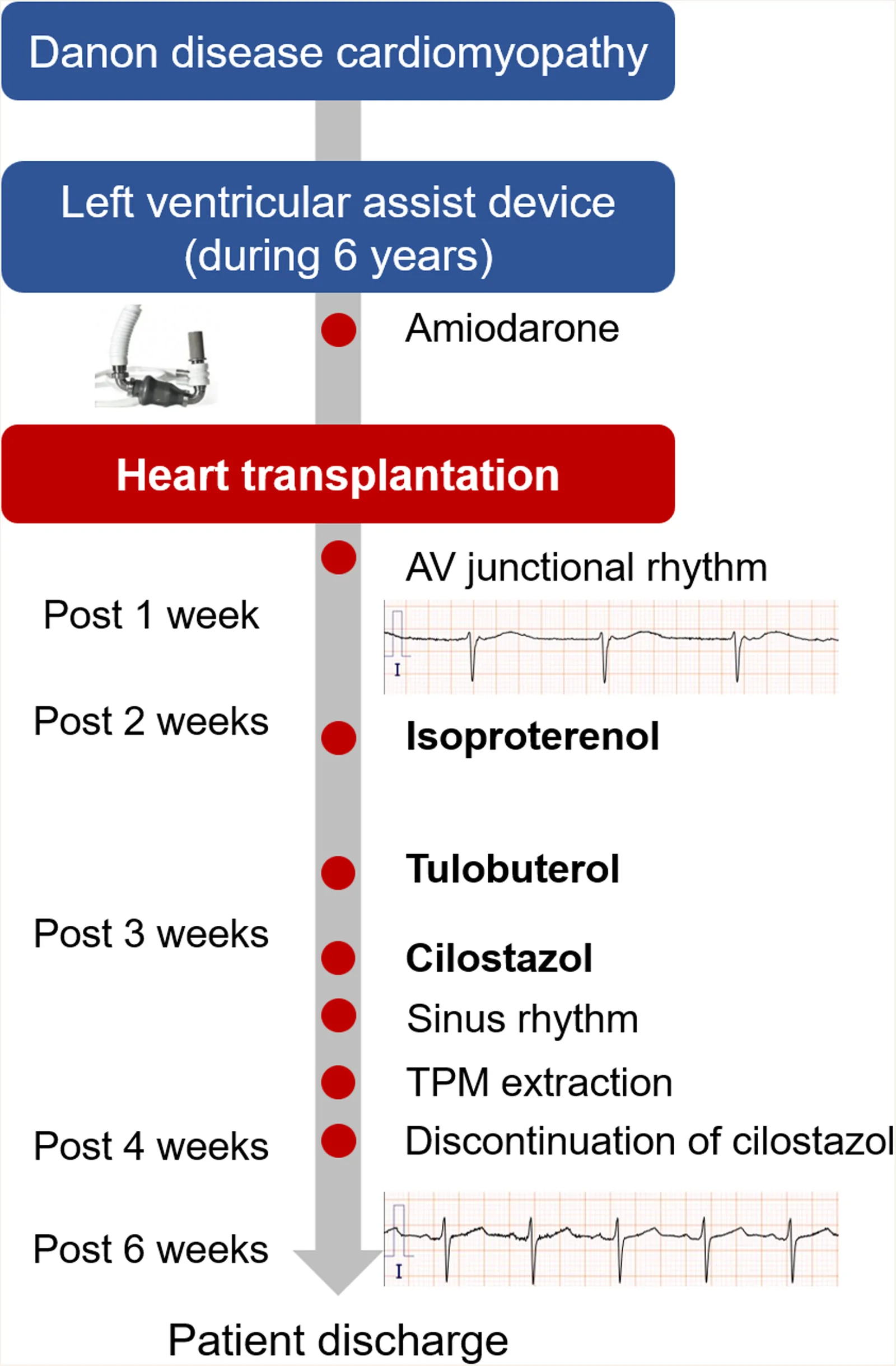
Adjunctive Cilostazol Therapy for AV Junctional Rhythm After Heart Transplantation AV = atrioventricular; TPM = temporary pacemaker.

## Conclusions

In this case, adjunctive cilostazol was temporally associated with improvement in HR and hemodynamic status in a heart transplant recipient with persistent SND. Cilostazol may be considered as a potential bridge before PPM implantation in selected patients, but further evidence is required.

## Funding Support and Author Disclosures

The authors have reported that they have no relationships relevant to the contents of this paper to disclose.
